# A qualitative study of men’s behavioural changes during weight loss maintenance

**DOI:** 10.1177/1757913920964516

**Published:** 2020-11-22

**Authors:** L Lozano-Sufrategui, A Pringle, D Carless, KJ Drew

**Affiliations:** School of Sport, Leeds Beckett University, Headingley Campus, 103, Fairfax Hall, Leeds LS6 3QT, UK; School of Human Sciences, University of Derby, Derby, UK; Centre for Creative-Relational Inquiry, The University of Edinburgh, Edinburgh, UK; School of Sport, Leeds Beckett University, Leeds, UK

**Keywords:** men, obesity, maintenance, weight loss, behaviour change, intervention, photo-elicitation

## Abstract

**Aim:**

This study aims to understand the behaviour changes men who attended a weight loss programme engage in during weight maintenance. Understanding the needs of men in the context of weight loss maintenance is important, as they are underrepresented in this body of literature.

**Method:**

Given its focus on personal experience, this study adopted a qualitative design. Semi-structured interviews supported by participant-generated photo-elicitation techniques to explore the behavioural changes 12 men engaged in 6 months after attending a men-only weight loss programme. Data analysis was undertaken through thematic analysis and Gleeson’s polytextual thematic analysis.

**Results:**

This study suggests that the key behaviours men engaged in to maintain weight loss can be classified into four categories: (1) ‘Small’ changes, (2) Informed decisions, (3) Monitoring of behaviours, and (4) Dealing with ambivalence.

**Conclusion:**

This study makes an original contribution to knowledge and can have important implications for practice in the area of men’s health, particularly with regard to the long-term impact of weight loss interventions.

## Introduction

In the UK, the rates of obesity have increased since the 1980s, and this rise is expected to continue. The majority of adults in England are overweight or obese, with more men (67%) than women (62%) being classified as such.^1^ Men are also more likely than women to experience obesity-related comorbidities. For example, the prevalence of diabetes is greater among obese men (14%) compared to women (11%).^1^ However, most men are underrepresented in hospital admissions related to obesity, in traditional weight loss interventions and in weight-related studies.^1,2^ To illustrate, in a recent systematic review on weight loss maintenance, 78% of participants were women.^3^ Similarly, in Garip and Yardley’s^4^ review of weight management studies, at least 224 out of 290 participants were women. Likewise, Pedersen et al.^5^ undertook a qualitative study to understand the complexity of self-regulating food intake in weight loss maintenance among short- and long-term weight loss maintainers. Their sample was also predominantly women, with only 4 males out of 18 participants. In addition, only 10% of patients receiving a primary care referral to commercial behavioural weight-loss interventions are men.^6^ However, current evidence and practice on weight loss and weight loss maintenance is still biased towards women’s needs.

Although little is known about men’s experiences of weight loss maintenance, there is a trend in the literature suggesting that weight regain is common after participation in weight loss interventions, with people regaining on average a third of the weight lost within a year.^7^ This suggests that regardless of gender, current obesity treatments are dissatisfactory in the long term, but little is known about the reasons for this.^8^ While previous studies have highlighted the behavioural issues that are important for long-term weight loss maintenance, such as self-regulation, problem solving and increased Physical Activity (PA),^9^ these intervention approaches are often just a replication or an extension of those used for initial weight loss.^3^ Furthermore, it is unclear how these intervention strategies are maintained in the longer term.

Given that masculinity is a culturally normative ideal of male behaviour, and it is constructed as the opposite of femininity,^10^ approaches to weight loss and weight loss maintenance should be designed with the needs of men in mind. Men’s perceptions of weight loss interventions as feminized may explain their underrepresentation in weight loss interventions.^2^ Promising evidence has demonstrated some intervention approaches that may be particularly appealing to men, such as the use of sports-based and homosocial environments.^11–13^ While these male-friendly approaches have been effective in increasing men’s uptake and adherence to weight loss interventions, understanding how men incorporate intervention strategies into their daily lives post intervention is still needed.^5^

With these thoughts in mind, this study aims to understand how men translated and incorporated intervention strategies and behaviour changes into the context of their daily lives in the 6 months after completing a weight loss intervention. In particular, this study explores how complex social, contextual, cognitive and emotional factors influence men’s attempts to maintain weight loss and health-related behaviour change in the longer term.

## Methods

This research is part of a larger project aiming to understand men’s health-related behaviours before, during and after attending a men-only weight loss intervention, called Tackling the Pounds [TtPs] (pseudonym). TtPs aims to help men lose 5% of their body weight in 12 weeks (one session of 90 min per week) through exercise and education. The findings of this study refer to men’s experiences of weight loss management during the 6 months after completing TtPs. Ethical approval was gained from the Research Ethics committee at Leeds Beckett University and by NHS REC [13/WA/0187]. Pseudonyms have been used throughout.

Given the focus of this study on understanding lived experience, a qualitative approach was used. According to Denzin and Lincoln,^14^ qualitative research is ‘a situated activity that locates the observer in the world. It consists of a set of interpretive, material practices that make the world visible’ (p. 4). This definition emphasizes the role of the researcher as an interpretive instrument that uses different practices to understand the life experiences of people. As qualitative researchers, we subscribe to the view of multiple realities, where reality is subjectively constructed by each person depending on context. Therefore, the philosophical assumptions guiding this study include a constructivist epistemology and a relativist ontology.

### Data collection

This study draws on semi-structured interviews and participant-generated photo-elicitation to explore men’s health behaviours in the 6 months following TtPs. Combining photographs with interviews was deemed an appropriate way of exploring the social, contextual, cognitive and emotional factors that influence weight loss maintenance, as this methodological approach can help generate vivid and personally meaningful accounts, some of which could have been overlooked through interviews alone.^15–17^

Participants were invited to provide or generate photographs for the specific purposes of this research. To do this, L.L.-S. contacted participants by phone to arrange a follow-up interview and she invited them to produce a personal photographic diary over a week about any behaviours they felt they had changed as a result of attending TtPs. They were asked to provide photos from their own personal archive or to take new photographs. Although L.L.-S. offered a disposable camera to take the photographs, all participants preferred to use their smartphones to complete this task. Then, they were asked to email L.L.-S. the photographs they had taken prior the interview. L.L.-S. printed the photographs and brought the hard copies to the interview, and/or participants brought photographs from their own personal archives to the interviews. During the interviews, L.L.-S. incorporated prompts to explore the participants’ visual and narrative-based accounts, such as: ‘Why did you take/bring this particular photograph?’, ‘What is this photograph showing and why does this matter to you?’, ‘In what context was this photograph taken and what makes it meaningful?’ and ‘What elements of this photo are related to your weight loss journey, and why?’. Data generated through discussion of visual materials were also supported by the use of a semi-structured guide, which explored (1) *the impact of the intervention on participants’ health behaviours*; (2) *barriers and facilitators for weight loss maintenance*; (3) *social support related to weight loss maintenance*; and (4) *participants’ motivation towards weight loss maintenance.* In total, 12 men agreed to participate in this follow-up phase of the research, and interviews lasted between 40 and 130 min.

### Data analysis

Data analysis aimed to capture both the verbal and the visual data. Therefore, analysis was guided by Braun et al.’s^18^ approach to thematic analysis and Gleeson’s^19^ polytextual thematic analysis. This means that while following the phases of thematic analysis proposed by Braun et al.,^18^ we assumed that both sets of data (verbal and visual) were linked. After transcribing each interview verbatim, each participant’s transcript and set of images was scrutinized for themes following an iterative process that involved moving back and forth from text to images. During analysis, L.L.-S. first familiarized herself with the data by conducting the interviews, listening and transcribing within 7 days of being produced, and making notes. After this, L.L.-S. started to develop themes by hand using a thematic map. Every time a new code was found, she added it to the map. Subsequently, she searched and identified potential clusters of patterned meaning on the mind map, which became tentative themes. These were then reviewed by the co-authors. Subsequently, the report was written.

### Participants

Twelve men agreed to participate in this study, all of whom completed the 12 sessions of TtPs. All participants were White British and self-reported being heterosexual, most (83%) participants had children and self-rated their health as ‘poor’ or ‘average’ (67%), of whom only a few (17%) ‘sometimes’ used health services. Ten participants reported maintaining the weight lost during TtPs and two (Milt and Lewis) continued to lose weight at follow-up. Information about participants’ sociodemographic profiles and health-related behaviours at baseline is provided in Table 1.

**Table 1:** Participants’ sociodemographic profiles and health behaviours at baseline

Name	Age range	Children	Employment	Reasons for attending TtPs	Baseline PA (times/week)	Self-reported health status	Use of health services	Weight classification
Matthew	29–39	No	Off work due to depression	Weight loss	Never	Poor	Never	Obese
Liam	70–79	3	Retired	Wanted a different exercise, control weight and maintain health	5+	Good	Never	Overweight
Charles	29–39	1	Unemployed	Weight loss and meet people	2	Good	General Practitioner (GP): sometimes Other health services: never	Overweight
Milt	40–49	No	Full time	His work colleagues were attending	2	Good	GP: often Other health services: never	Overweight
Abraham	60–69	1	Retired	Weight loss	2	Good	GP: often Other health services: never	Overweight
Conan	60–69	3	Retired	Diabetes, weight loss, mobility	3	Average	Never	Obese
Ralph	60–69	3	Retired	Get fitter, weight loss, improve health	3	Average	Never	Obese
Lewis	50–59	2	Full time	Improve health and self-esteem	Never	Average	Sometimes	Obese
Doyle	40–49	3	Unemployed	Improve health, weight loss, feel better	3	Average	Never	Obese
Ian	40–49	2	Unemployed	Weight loss, meet people, improve confidence	4	Average	Sometimes	Obese
Bert	60–69	2	Retired	Improve health	5	Average	Sometimes	Overweight
Malcolm	29–39	3	Full time	Work colleague attending	2	Average	Never	Overweight

### Researcher’s reflexivity

In line with the constructionist epistemology that underpins this research, the accounts shown in this study are a result of a process of interaction between participants and L.L.-S., who collected the data informing this study. The following paragraph, written in the first person by L.L.-S., shows how her own subjective experiences may have shaped this research.

*am a heterosexual female with an athletic body shape. My BMI is 20* *kg/m*^2^ *and I am a long-distance runner. I am White, middle-class and from Spain, so English is not my mother tongue. I was 28* *years old at the time of data collection. I believe that my gender, nationality, body shape and age influenced my interactions with participants, the information they shared with me and their engagement in this research. For example, participants did not talk about intimacy with me, even though male authors found that sexual frequency increases among men post weight loss.*^20^ *Furthermore, due to a limited linguistic and cultural understanding, I was unable to participate in gendered talk such as jokes and banter. To overshadow this limitation, I engaged and excelled at other gendered social practices during the sessions, such as football and some exercises such as push-ups, where I tried to achieve the highest number of repetitions within the group. According to Alexander et al.*,^21^ *banter is integral to social cohesion among British males, and it allows men to bond with each other. It also helps facilitate discussions of sensitive topics such as weight and health.*^11^ *For these reasons, I had to find other acceptable ways of building trust and rapport with participants, such as showing a caring attitude, giving them information about myself and my country when requested (i.e. participants were particularly interested in my country’s football teams and traditional food), and making sure I participated in the activities when they invited me to do so, instead of intruding at times when I may not have been welcomed.*

## Results

In total, five men used visuals to support their talk. Conan took two photos about food; Milt brought 10 photos to the interview, about the weather, exercise, snacks at work, illness and Do It Yourself (DIY) activities; Abraham provided 14 photos, all of them about food; Ralph brought photographs of his car, his grandchildren and the hospital; and Lewis was the only participant bringing photographs to the interview that would enable him to compare his lifestyle pre TtPs with the behaviours he engaged in during weight loss maintenance. The most common reasons participants gave to explain their lack of participation in photo-elicitation included: (1) a preference to rely on verbal accounts, (2) the extra perceived burden of taking photographs, and (3) lack of confidence using this methodology. Findings from verbal and visual data can be categorized in four key themes: (1) ‘Small’ changes, (2) Informed decisions, (3) Monitoring of behaviours and (4) Dealing with ambivalence.

### ‘Small’ changes

Participants stressed the importance of adopting small lifestyle changes for long-term weight loss maintenance, and they developed their own strategies to fit in PA and healthy eating within their daily lives and identities. These strategies related to the introduction of active ways of living and the consumption of healthier foods.

#### Active living

**Figure 1.**
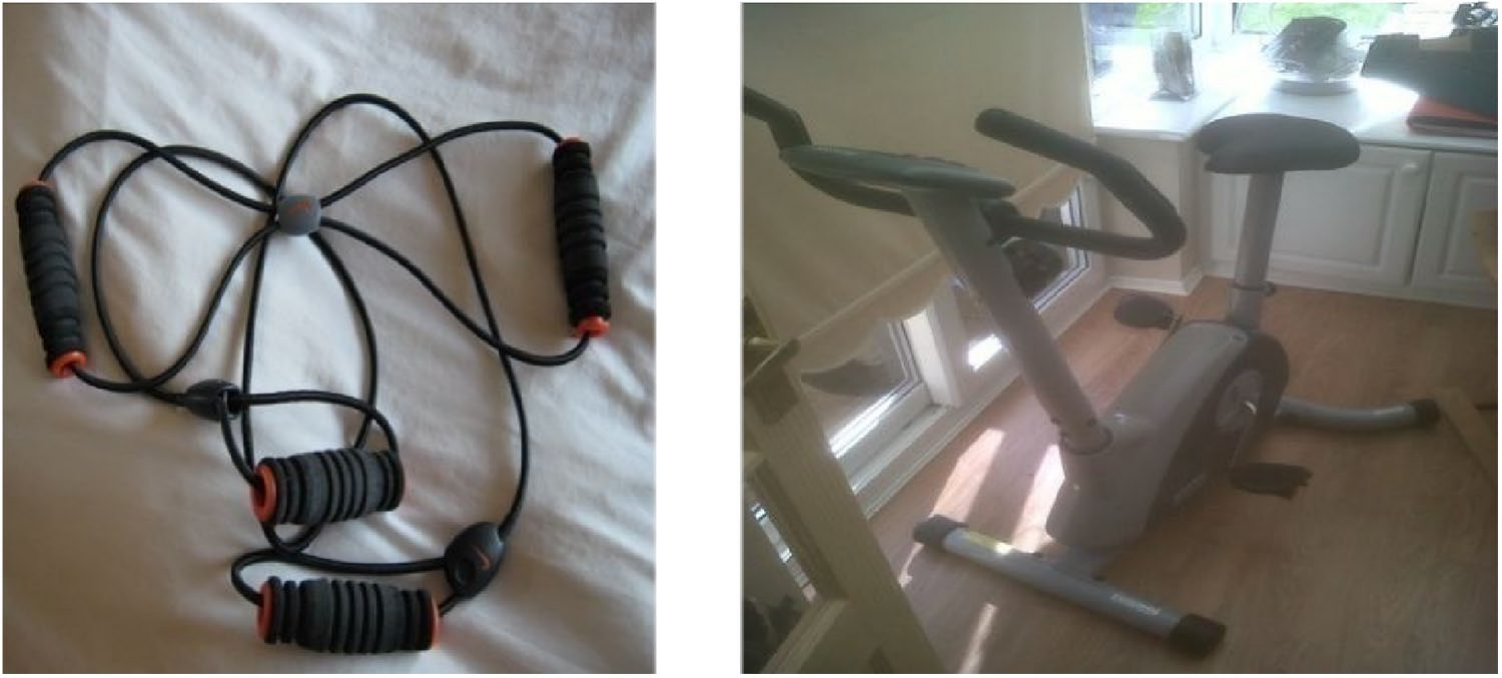
Exercise bike.

While increasing daily PA can be difficult for people with multiple commitments (e.g. work and family), participants used strategies to increase PA without this compromising other commitments. For example, some participants reduced their sedentary behaviours by introducing active ways of doing activities that are typically sedentary, such as TV watching and reading. In some instances, these changes resulted from a process of transferring lessons from TtPs into creative responses in daily life. Figure 1 and Lewis’ example below show how TtPs reached beyond its immediate spatial confines after 4 weeks:

Lewis: Extra work equals extra benefits, so I’ve got my own stretch bands, so I can do some of the exercises that we did with [lifestyle coach]. And me exercise bike, which lives in the conservatory […]

Researcher: Did you have this before TtPs?

Lewis: I had that yeah, and it was used as a clothes horse. Basically, [wife] used it to dry the washing on it, because that’s all the use it ever got.

Researcher: When did you start to use it?

Lewis: I started to use it probably after a month, when I had been here [TtPs] for about four weeks, and I was beginning to get used to the level of physical activity and I was seeing some benefits from losing weight as well, and I thought, well, why not, give it a go […] so I am there and my wife is here [pointing at the photograph of the bike], and we can still have a conversation, still watch the TV and get generally warm, a bit of sweat on and then go and have a shower and exercise done.

By suggesting ‘we can still have a conversation, still watch the TV’, Lewis is implying that the incorporation of more active ways of living in his daily routine is only an addition to what he used to do, not a disruption. Other small lifestyle changes some men introduced in their daily lives to increase their PA levels included, for example, walking instead of driving. These changes brought about several benefits beyond increased PA levels and fitness, such as saving money:

Researcher: Do you have any examples of the things that you do now that you didn’t do before?

Charles: So if I am going into town then I won’t park in the middle, I’d park at a cheap car park that is further into town and walk into town and I wouldn’t get the lift or the escalators, I would take the stairs. I walk to football if I go to the stadium, even though is 45 min walk. It’s probably just as long in the car with all the traffic, so I’d take that, I’d rather walk again just listen to my music and so it saves money, it’s a better exercise.

**Figure 2.**
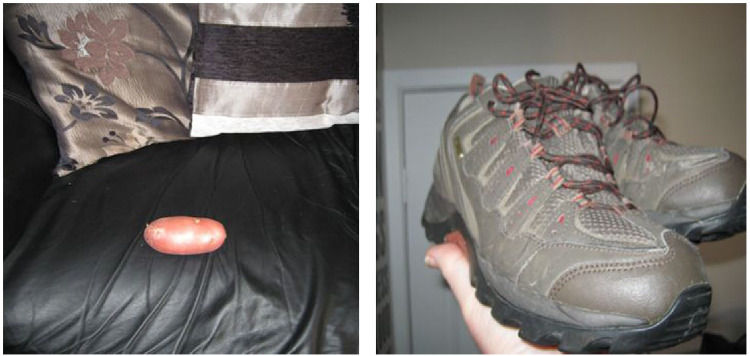
Coach potato versus walking boots.

Besides reducing sedentary time and increasing PA at home, Lewis also talked about having active breaks during work. After showing the following photographs (Figure 2) to illustrate his reduction of sedentary time and increase of PA, he said:

Lewis: That’s me coach potato [laughs] so I am not sat there watching the TV anymore, and now I’ve got me walking shoes. I spend a lot of time in those […] I don’t just kind of walk around the home. I took the opportunity when I’ve been out and I’ve been working, in my lunch hour, I’ll maybe get my rucksack, and I’ll get my bag, pop me sandwiches in the bag, change into my walking shoes, park the car and maybe walk 25-30 min up the hills, sit down up the hills, have my lunch, and walk back down. So it’s a break in the day, it breaks the day up for me, and it gives me that added bit of boost.

#### Healthy eating

Participants adhered to healthy eating habits during weight loss maintenance. This process was not always straightforward, and involved breaking with some ‘bad habits’, as Malcolm explained:

*I got into a routine where I was eating quite a lot. I had quite big plates. But now my portion sizes are similar to probably one of my kids, to be honest […] The hardest part for me was leaving food, if I felt full. I was always brought up I had to eat the whole plate, and I got into that routine. I’d eat my plate, and if the kids left theirs, I’d eat theirs. But now, if I am full, I’ll stop, I’d actually leave food. That was a difficult lifestyle change I made, because I don’t like seeing waste, so I wanted to finish it.*

**Figure 3.**
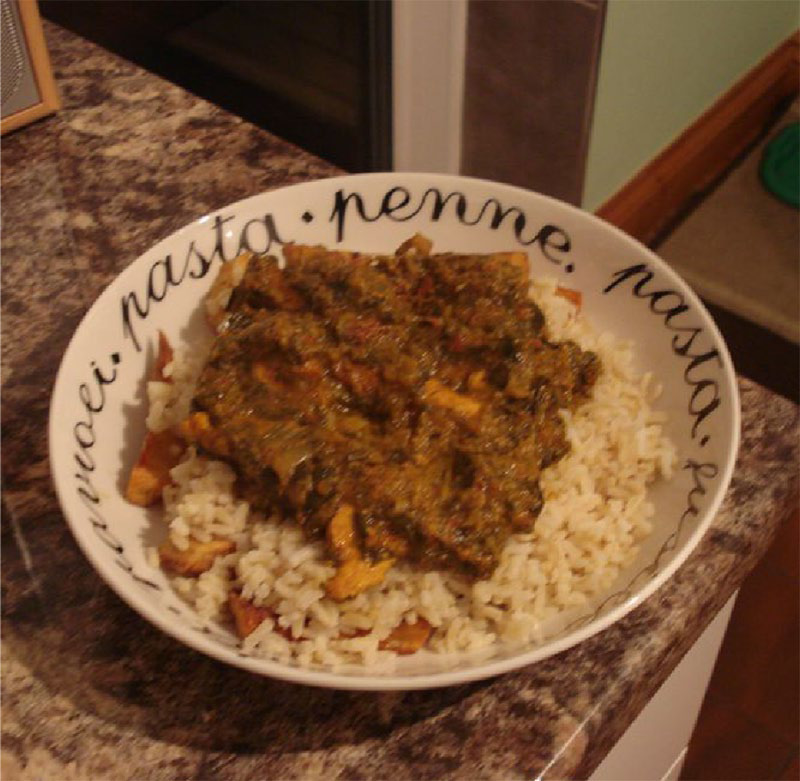
Portion sizes.

Arguably, during the intervention, men like Malcolm learned to bring overeating back into consciousness, and reflected upon their unconscious tendency to ‘eat the whole plate’. Echoing this point, and showing a picture of one of the meals he made (Figure 3), Abraham said:

*Before coming to [TtPs], on a Saturday night, I used to make a curry and rice, and we’d eat all of it. But now probably because of TtPs we cook the same amount, we eat probably half of it, and save the rest.*

Being consciously aware of ‘how much’ extra food was being eaten not always resulted in an increased self-control of eating behaviours. In many cases, structural changes were needed, as explained by Milt:

Researcher: Do you think you have control over your intake?

Milt: I think if you gave me a big table full of food, there’s a bit of a programme on me that would eat that, that could eat that, so the only thing is I’ve gotta find ways of not doing that, like using a smaller spoon, slowing down the eating, things like that.

From this, it appears that mindful regulation of food intake was difficult for participants. Hence, Milt changed his food environment to more effortlessly manipulate his consumption in a less disciplined way, which facilitated his adherence to weight loss maintenance. Some men also talked about cutting *down*, instead of cutting *off*, the pleasurable unhealthy foods they still enjoyed:

Researcher: Does your diet now differ from what it was before coming to TtPs?

Ralph: Yes. I would say I am eating healthier than I was. I am not saying it’s perfectly healthy, but I would say it’s a lot more of a healthy diet than it was. And especially lately, I’ve gone back into the salads again, and I am quite enjoying the salads. But my favourite has always been chips, and that’s been my downfall, these chips. I mean I can eat them no matter where I go, it doesn’t matter. But I do try now to not have them like three or four times a week, in fact to be honest with you, I may be lucky if I get them once a week these days.

### Informed decisions

**Figure 4.**
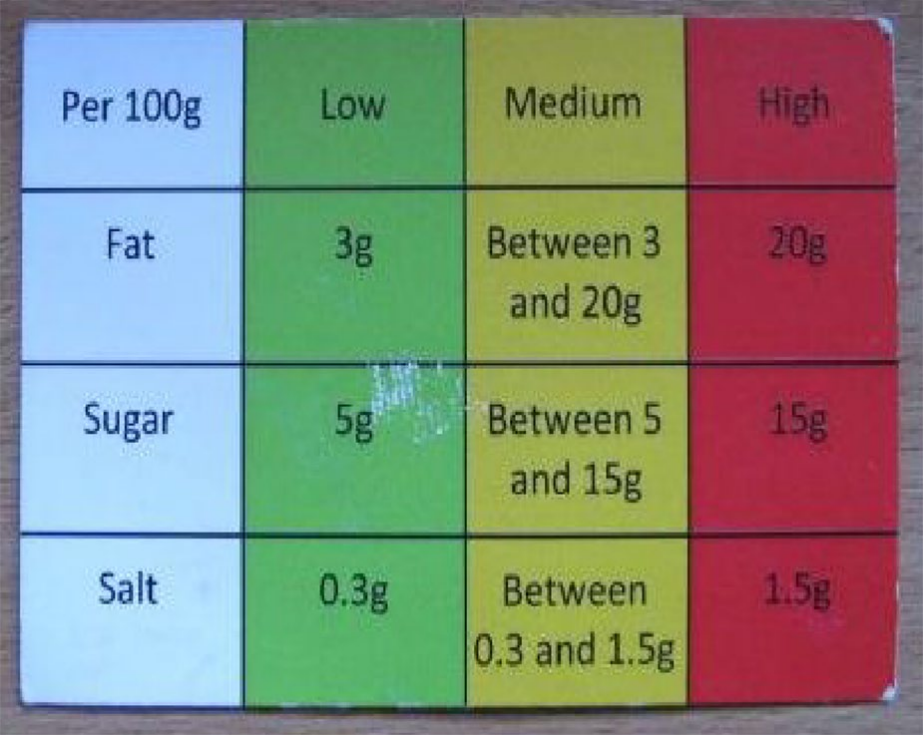
Traffic light guide.

In this study, some men used the simple and tailored supportive materials they were given during TtPs. These resources facilitated men’s attempts to become active agents in their health choices. Many of the weight loss maintainers referred to a traffic light shopping guide that was delivered to them during TtPs. This tool provided flexible guidance for food choices, and it was central to men’s sense of autonomy when making decisions about what to buy. Lewis took a photograph of it (Figure 4) and described it as his new ‘best friend’:

*This is my best friend, the fella that comes shopping with me, and he makes a big difference to what goes in the basket. So I read labels now, I drive my wife mad, ‘What are you doing, just put it down!’ ‘No, no, hang on a minute, I just need to have a look! No we don’t want that one, we want this one’. But yeah, I am very conscious about what to buy and what I put in the basket and what we shop for […] I look at the low content, I look at the low one, trying to look at the green one if I can. If it’s in the red, then I don’t buy it at all. If it’s in the yellow, it is acceptable. But my preferred choice would be to go through the green, always.*

For Lewis, foods that fit in the green column are *good* foods, whereas he avoids foods that are in the red column, because they are *bad*. This easy-to-do evaluation of the goodness of different types of food helped participants transform complex data into meaningful information. As a result, the cognitive effort required to make healthy choices was lowered, and the simplicity of approach like this may contribute to maintaining long-term health behaviours.

### Monitoring of behaviours

Participants used different approaches to keep track of their progress towards their health-related goal(s). Some of these included using mobile apps, spreadsheets, self-monitoring of weight and experiential feelings, particularly with clothes. ‘Visualizing’ and ‘feeling’ weight changes enabled the men to bring habitual behaviour into conscious awareness, and these became key mechanisms of self-surveillance. For example, Conan talked about a spreadsheet, which he designed to monitor his weight loss/gain across time:

Conan: I keep a tab of how often I’ve been to the gym, what exercises I’ve done, what I’ve achieved, that sort of thing. I keep a sort of record in my book and on a spreadsheet.

By deliberately self-recording his weight over time, Conan portrays a type of self-regulation style that could help him control his weight. Although other men did not record their weight as thoroughly as Conan, they became more aware of experiential cues such as assessing the tightness of their clothes. These cues were less premeditated than self-monitoring, but still provided relevant information. Milt demonstrated such a self-regulatory approach:

Researcher: Do you have any specific targets in terms of weight loss?

Milt: Yeah. The trousers I’ve got on now are size 40, up until a few years ago I was in a 38, and I’ve been able to tighten the belt for the last few months, and I am almost where I can get to a size 38. So I think I wanna be able to get into a size 38, I mean once I get there obviously the next goal. But I heard somewhere, for a man, if you are a 40 inch waist you are much more likely to get diabetes […] So that’s probably, that’s my ultimate aim. It’s like each Monday when I put my work pants on, it’s like, are they tight, or are they comfortable?

### Dealing with ambivalence

Dealing with ambivalence involved men making decisions between what is thought to be *good* according to a medicalized regime of self-control, and the men’s personal health needs. In this process, men became reflexive subjects who were ‘willing to undertake self-reform but at the same time rationally manage the experiential conflicts produced by that reform’ (p. 231).^22^ In this context, the consumption of the occasional treat was normalized, as explained by Matthew:

*I’ve noticed it myself really, I do like to for four to five days eating healthy, pasta, chicken, broccoli, etcetera, I just need that sugar rush in me, because I’ve not had it for a bit. But as soon as I’ve had that sugar rush, I can’t stop, and that’s when… one and a half days, two days, I think, I need to stop. I need to get to that gym and get it all out of me, then I’ll carry on eating healthy, etcetera, and then I may go to 5 days, 6 days, and then I need another boost of sugar.*

Additionally, Liam, whose wife had a stroke a few weeks before the interview, mentioned drinking as a coping strategy. However, he realized that he was not ‘going anywhere’, and he planned to reduce his alcohol consumption to a limited number of days:

Liam: I don’t think I will ever say I will never drink again. Because there are occasions when it’s social and I enjoy it. So, I think my target would be to increase alcohol-free days […] Like not touch alcohol Monday, Tuesday, Wednesday and Thursday, but you can go out for a couple of pints Friday and maybe one on Saturday. So you are actually reducing your units, but not denying yourself the complete pleasure.

Others learned how to compensate for unhealthy behaviours: for example, Abraham adjusted his health behaviours as follows:

Researcher: What did you do last week that you think has helped you to lose one kilo?

Abraham: Nothing out of the ordinary really; I just carried on with the same regime. And I am quite happy this is working, I don’t, I’ve not, I mean say we went to a wedding and that: ‘Oh God, I did knock a few back there and I had a big meal’, you know what I mean? But I thought: ‘Well, I’ll get it off when I get back’, you know? That type of thing. ‘Coz you don’t want to go backwards, you know, you’ve got that goal when you do set your mind to it.

Some men were able to make ‘personal assessments of risk’, meaning that instead of conforming to a medicalized regime of self-control, which depicts those who do not conform to its logic as ‘ignorant’, ‘apathetic’ and ‘lacking self-efficacy’; participants (un)consciously transgressed biomedical interdictions by enacting independence and autonomy:

Researcher: Did you learn anything in TtPs about fizzy drinks?

Abraham: Oh yeah, I understand what they are saying, I accept everything what they are saying but I don’t know, I seem to want to break the rules if you know what I mean, because I don’t want to go down such a path where I think people’s got getting to a situation where they say: ‘Oh I would love to eat that but I can’t’. You know I shouldn’t eat that. I am not going down that avenue. I don’t want to do that, if I want to eat it I’ll eat it.

Despite coming across as transgressive, this does not mean that Abraham was careless about his health. For him, subordination to a medicalized regime of external control, which did not account for his personal needs, undermined his self-control and was alienating. As a result, he developed his own mechanisms of self-control, as this approach was more aligned with his identity. Charles also talked about deploying portion control strategies as a way to behaviour change:

*I used to go and buy a packet of biscuits, I would just sit there in front of the TV and eat the biscuits and eat lots of them. But now I’ve got a biscuit barrel and I say to her [girlfriend]: ‘Take three biscuits out of the barrel, have them with a cup of tea, and then that’s it’. And the barrel stays there in the kitchen, and it’s easier, a pack of biscuits lasts two weeks now, whereas before lasted two days at the most.*

## Discussion

The uniqueness of this study lies in its capacity to understand the behaviours that men engage in to maintain weight loss after completing a weight loss intervention. This knowledge matters because men have been unfairly underrepresented in this area of research. Besides the sample being unique, the use of visual methods in this study is also innovative in an area where most qualitative studies have privileged verbally transmitted knowledge. The combination of interviews with photo-elicitation methods enabled some participants to better express themselves and illustrate resources for engaging in long-term behaviour change, thus allowing for the emergence of ‘localized understandings’ (p. 685)^17^ of weight loss maintenance. In doing this, this study shows how men incorporate behavioural changes into their lives and accounts for the cognitive, emotional and contextual influences on weight loss maintenance.^3^

The knowledge generated in this study has important implications for practice, as it can be used to support the development of effective weight loss interventions for men. This includes both the design of interventions to help men lose weight and the implementation of strategies to help men maintain weight loss in the long term. This is important because most intervention approaches to maintenance replicate those used for initial weight loss^3^ and, unsurprisingly, often fail.^7,8^ The findings of this study have highlighted the different types of behaviour change strategies that are feasible for men to apply in the context of their lives to sustain weight loss after attending a weight loss intervention and include: maintaining small changes that do not disrupt with the normal course of their lives; using simple tools to guide their decisions about diet; developing own strategies to control portion sizes; and monitoring of behaviours and having a healthy lifestyle while still accounting for their own personal needs. This information can be used by policy makers and practitioners to design and implement interventions that are not only successful but also sustainable in the long term, even post intervention.

This study adds new insights to the literature on masculinities and health. It suggests that, far from being unconcerned about their health, men who attended a weight loss intervention rely on a complex web of skills to maintain healthy lifestyles. While it has been suggested that women are the ‘experts’ in the feminized realm of dieting – a feminine activity that is about looking slim and pretty, linked to vanity,^10,23^ men also have a degree of expertise comparable to women when it comes to weight loss maintenance. Participants in this study showed an ability to make rational and informed decisions about their behaviours, which favourably contributed to weight loss maintenance. However, instead of adhering to a medicalized regime of self-control, many men talked about the strategies they had developed to *be healthy* in a way that was aligned with *their identities*. This is a new insight in the literature on weight loss maintenance and suggests that men *can* and *do* change their behaviours during a weight loss intervention, but the successful maintenance of these internalized behavioural regulations are more likely to occur when these changes are in harmony with the normal development of their lives. Instead of internalizing neoliberal constructions of the responsible healthy citizen, men in this study prioritized their own identities over other societal pressures.^24^

Given that discussions on obesity, dieting and weight loss are related to our ideas about beauty, normality and acceptable behaviour,^8^ these findings provide a reflection of modern society. It is interesting to note how the findings from this study differ from studies on weight loss dominated by, or exclusively focused on, female samples. Many of these studies report participants feeling a tension between the obese past and the present day and used negative words like ‘battle’ to refer to their weight loss maintenance efforts.^8^ Additionally, in relation to diets, previous studies have found that sustaining diets was experienced as a state of ‘effortful vigilance, requiring considerable personal strength and commitment’,^25^ but again these were dominated by female samples. However, there seems to be a different type of societal pressure on women’s bodies compared to men’s, which inevitably influences how women and men experience weight loss and weight loss maintenance. Men in this study did not use those war-like, negatively framed words and moral overtones when discussing behaviour change. For them, flexibility and enjoyment were the key to success.

While this study makes a significant and original contribution to the literature on weight loss maintenance, it is important to highlight some cautionary notes when interpreting these results. Most of the theory used to underpin our understanding of participants’ experiences of weight loss maintenance draws on psychological understandings of health behaviours. While we do not see this as a limitation, we believe that understanding the complexity of human life is perhaps out of reach of psychologically orientated approaches. Thus, there remains a need to draw on sociological thought, in particular with regard to health practices to understand how men are able to negotiate and navigate through the sociocultural health world. This could help understand why interventions like TtPs help some men at certain times of their lives, but not others.

Some lessons can also be learned from the use of photo-elicitation techniques with men in the area of weight management. In this study, only 5 out of 12 men used visuals to support their talk, meaning that most participants preferred to privilege verbally transmitted knowledge. This is, in itself, an important methodological finding, as it problematizes and challenges the idea that men are verbally inexpressive.^26^ Some of the reasons mentioned by some participants for not providing photographs also suggest that clear instructions and appropriate support is required when undertaking qualitative studies using visual methods.

Given the homogeneity of the sample in terms of ethnicity and sexuality included in this study, future research should explore the experiences of weight loss and weight loss maintenance among other groups of men, such as gay men, men from ethnic minority groups, as well as the experiences of those men who do not complete interventions. Furthermore, since the body is a core part of the reflexive project of self-identity,^27^ future research should also investigate the impact behaviour change and weight loss may have on men’s identities. This call for future studies was already proposed nearly two decades ago by Sarlio-Lähteenkorva,^8^ but it is still an underexplored area.

## Conclusion

Although more men than women are overweight or obese in the UK, and more likely to experience obesity-related comorbidities, current practice on weight loss interventions and weight loss maintenance is biased towards women’s needs. Furthermore, weight regain is common after participation in weight loss interventions, suggesting that the current obesity approaches are dissatisfactory in the long term. For these reasons, this study aimed to understand how men who attended a weight loss intervention incorporate intervention strategies within the context of their lives post-intervention.

Adopting a qualitative approach which drew on semi-structured interviews and participant-generated photo-elicitation, this study explored men’s health behaviours in the 6 months after attending a community-based men-only weight loss intervention. As observed in our study, the behavioural changes men implemented in the weight loss maintenance phase were aligned with their identities and fitted within the context of their lives. The ‘localized understandings’ of weight loss maintenance we have found in this study have been possible due to the use of innovative methods of data collection, such as participant-generated photo-elicitation and sophisticated methods of analysis, including a combination of Braun et al.’s^18^ thematic analysis and Gleeson’s^19^ polytextual thematic analysis.

According to the men interviewed, maintaining weight loss was a result of introducing changes that were small and fit within the contexts of their lives and identities, such as active living and healthy eating; being able to make informed decisions by using the information and tools provided and learned within the intervention; adopting different strategies to monitor their health behaviours and weight; and developing personal strategies to deal with ambivalence, by allowing themselves treats and compensating for unhealthy behaviours.

Our research has shown the ways in which intervention strategies that support weight loss maintenance may differ from those that promote initial weight loss, particularly among men. Notably, interventions could promote long-term effectiveness if they account for the context of people’s lives, for example, by identifying and addressing the cognitive, emotional and contextual factors that influence people’s attempts to maintain weight loss. Additionally, our study has shown the complex web of skills men rely on to maintain healthy lifestyles. This knowledge may help policy makers and health practitioners to design and implement interventions that not only lead to successful and sustainable outcomes but could also help address the unique health needs of a population group that has traditionally been overlooked in this area of research and practice.
